# Investigation of the Prevalence and Diagnosis of Chronic Obstructive Pulmonary Disease in a Group of Elderly Individuals Residing in an Island Area of Ningbo

**DOI:** 10.1155/2019/6918340

**Published:** 2019-08-01

**Authors:** Wei Sheng, Youchang Huang, Zaichun Deng, Hongying Ma

**Affiliations:** ^1^The Affiliated Hospital of Medical School of Ningbo University, Department of Respiratory Medicine, Ningbo 315020, China; ^2^Hepu Town Central Hospital, Xiangshan, Ningbo 315733, China

## Abstract

**Objective:**

This epidemiological investigation aimed at determining the current situation regarding the diagnosis and treatment of chronic obstructive pulmonary disease (COPD), especially missed diagnosis and missed treatment, in a group of individuals residing in an island area of Ningbo.

**Methods:**

Adults ≥60 years of age were selected from an island area of Ningbo. All participants completed a COPD-Screening Questionnaire and underwent a post-bronchodilator pulmonary function test. COPD-positive individuals then completed a questionnaire surveying the status of diagnosis and treatment of COPD and the reasons for missed diagnosis and treatment. The data were collated and analyzed using SPSS version 22.0 (IBM Corporation, Armonk, NY, USA).

**Findings:**

(1) A total of 1526 individuals were screened, of whom 1371 (89.8%) were eventually included in data analysis. From these, 254 were diagnosed with spirometry-defined COPD, corresponding to an overall prevalence of 18.5%. Prevalence was higher in men (28.9%) than in women (8.3%) among the sample. (2) According to chi-squared test results, risk factors for COPD included sex, age, smoking history (pack-years), cough, and dyspnea. Body mass index, family history of respiratory diseases, and exposure to biomass smoke from cooking were not risk factors for COPD. (3) Multivariate logistic regression analysis revealed that age and smoking were independent risk factors for COPD. (4) Receiver operating curve analysis revealed that, at a cutoff of 19.5, the highest sum of sensitivity and specificity was 69.7% and 75.5%, respectively. The COPD-Screening Questionnaire could be used as a preselection method to screen for COPD in primary care settings. (5) Of 254 individuals diagnosed with COPD, only 10 had a history of COPD and only 35 had a previous diagnosis of pneumonia or bronchitis. These data revealed that the rate of missed diagnosis of COPD in the Ningbo island area was 96.1%.

**Conclusion:**

The prevalence of COPD among elderly individuals in the Ningbo island area was significantly higher than in other parts of China. Moreover, the rate of missed diagnosis of COPD in the Ningbo island area was extremely high. Smoking and age were independent factors for the occurrence of COPD.

## 1. Introduction

Chronic obstructive pulmonary disease (COPD) is a disease characterized by limitations in reversible airflow. It is also a common disease that can seriously endanger human health [1]. The Global Burden of Disease study estimated that 174.5 million adults were affected by COPD, with a disease burden of 384 million in 2015. It was estimated that 3.2 million individuals died of COPD that year [2, 3]. By 2020, COPD is projected to rank fifth among the world's economic burdens of disease and third in global causes of death [4].

The China Pulmonary Health (CPH) study [5] reported that the prevalence of COPD was 8.6% in individuals ≥20 years of age, corresponding to approximately 99.9 million Chinese adults. The prevalence of COPD was 13.7% in individuals ≥40 years of age. According to the age group, the prevalence of COPD was 21.2% in individuals 60–69 and 35.5% in those ≥70 years of age. By comparing the prevalence of COPD in those ≥40 years of age, the prevalence rate reported by Wang Chen (13.7%) is significantly higher than the rate reported by Zhong et al. (8.2%) [6]. These data indicate that the prevalence of COPD has increased yearly in recent years, especially in those ≥60 years of age. The island area is a special remote area. Due to insufficient knowledge of COPD, low levels of education, relatively low level of health care, and insufficient availability of medical equipment, such as pulmonary function meters, the diagnosis and treatment of COPD in rural areas is unclear. There have been few epidemiological investigations of COPD in island areas domestically and abroad, and investigations examining the prevalence and missed diagnosis of COPD in individuals ≥60 years of age are relatively insufficient. Therefore, we aimed to estimate the prevalence of COPD in the Ningbo island area among individuals ≥60 years of age, as well as its rate of diagnosis and treatment. Furthermore, we gathered and assessed risk factors relevant to COPD and the level of awareness of the disease.

## 2. Outcomes

### 2.1. Study Participants

**Figure 1:**
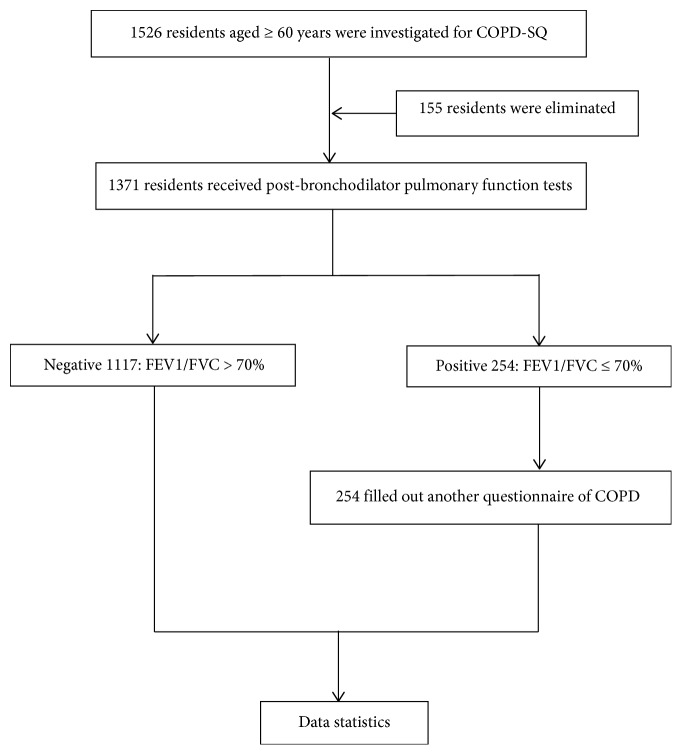
Flow of participants through the study.

Individuals ≥60 years of age, who resided in several villages in Hepu Town, Xiangshan County, Ningbo City, were investigated from December 2017 to August 2018 (Figure 1). Those with acute respiratory infection in the past month, severe heart disease or other pathological changes in the lungs (such as bronchial asthma), decreased comprehension, dentures, or other conditions that were not amenable to pulmonary function tests, were excluded.

## 3. Methods

### 3.1. Research Method

A census method was used. All participants completed a COPD-Screening Questionnaire (COPD-SQ) and underwent a post-bronchodilator pulmonary function test. The COPD-SQ consisted of seven items that are as follows: age, smoking pack-years, body mass index (BMI), cough, dyspnea, family history of respiratory diseases, and exposure to biomass smoke from cooking [7].

### 3.2. Detection Method

An automatic lung function meter (CareFusion MicroLab) was used. COPD was defined as a post-bronchodilator forced expiratory volume in 1 s (FEV_1_)/forced expiratory volume (FEV) ratio <0.7, according to the 2017 Global Initiative for Chronic Obstructive Lung Disease (GOLD) guidelines [8]. The ratios of observed to predicted FEV_1_ were also calculated according to GOLD guidelines and used to stage the degree of obstruction (GOLD stage I, ≥80% predicted; GOLD stage II, ≥50% to <80% predicted; GOLD stage III, ≥30% to <50% predicted; and GOLD stage IV, <30% predicted) [9]. Each participant agreed to the content of the examination and obtained the final test results.

### 3.3. Scoring

Individuals who were positive for COPD completed a questionnaire surveying the status of diagnosis and treatment of their disease and the reasons for missed diagnosis and treatment. Survey contents addressed education level, occupation, smoking and smoking cessation, COPD Assessment Test (CAT) [10], modified British Medical Research Council Dyspnea Rating Scale (mMRC) [10], highest hospital, highest medical department, symptoms, history of other diseases, number of acute exacerbations, reasons for nonconsultation, and medication, among others. The CAT questionnaire included a total of 8 questions surveying cough, expectoration, chest tightness, climbing or climbing stairs, housework activities, confidence in leaving home, sleep hygiene, and energy. Each question was based on the severity of the disease. For 0 to 5 points, the patient answered each question and the investigator calculated the total score after scoring each question. The CAT score ranged from 0 to 40 points. The mMRC score was determined using the mMRC scale to determine the severity of the patient's dyspnea. Level 0 was generally smooth when breathing autonomously; in Level 1, there was shortness of breath when walking fast or going uphill; in Level 2, normal walking speed was slower than that of healthy individuals. Walking at regular speed on flat ground induced obvious shortness of breath and the need to stop to breathe; in Level 3, walking 100 m or a few minutes on flat ground would induce difficulty with breathing and the need to stop to breathe; and, in Level 4, there were obvious breathing difficulties, even when calm, and no task could be performed.

### 3.4. Statistical Methods

SPSS version 22.0 (IBM Corporation, Armonk, NY, USA) was used for statistical analysis. The chi-squared test was used to compare the prevalence of various risk factors, while multivariate logistic regression analysis was used to analyze the influence of these factors; *P* < 0.05 was considered to be statistically significant.

## 4. Survey Results

### 4.1. General Information

**Figure 2:**
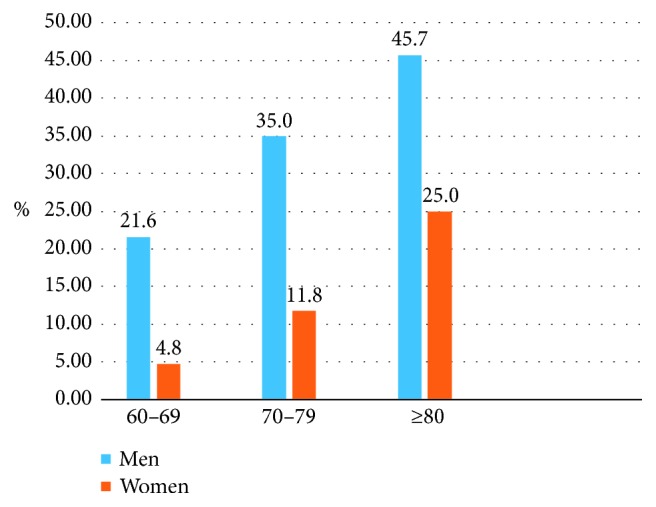
The prevalence of COPD in different age groups and sex groups compared in a column chart.

- The total sample size was 1526; however, 155 individuals who did not meet the requirements were excluded, thus leaving 1371 who were enrolled, including those who completed the questionnaire and passed the lung function test, corresponding to an enrollment rate of 89.8%. The prevalence of each factor was investigated, and the findings are summarized in Table 1.
- The prevalence of COPD was different according to sex and age (Figure 2).
- The relationship between various factors and the prevalence of COPD.
- The chi-squared test revealed that the prevalence of COPD was different according to sex, age, smoking, smoking pack-years, cough, and dyspnea (*P* < 0.05); the difference was statistically significant. The prevalence of COPD among individuals with different BMI, family history of respiratory diseases, and exposure to biomass smoke from cooking was compared (*P* > 0.05), and the differences were not statistically significant.

### 4.2. Multivariate Analysis of the Prevalence of COPD

The abovementioned univariate analysis revealed that sex, age, and smoking were statistically significant for the diagnosis of COPD and were used as independent variables to determine the presence of COPD. Multivariate logistic regression analysis revealed that age and smoking were independent risk factors for COPD (Table 2).

### 4.3. COPD-SQ

The average COPD-SQ score of those enrolled in the present study was 17.4 ± 5.2; however, the average COPD-SQ in those with COPD was 22.2 ± 5.4.

**Figure 3:**
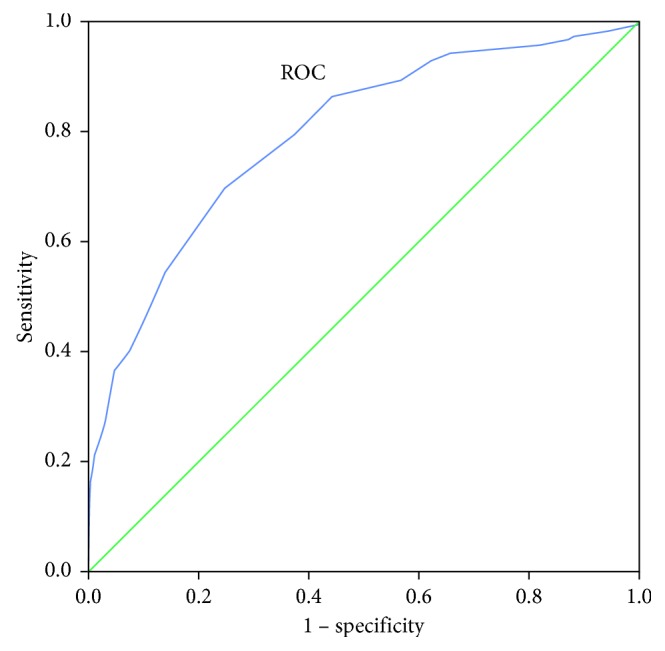
The ROC curve of COPD-SQ.

When the area under the ROC curve was 0.795 (*P* < 0.01), it indicated that the COPD-SQ could be used to screen for COPD in the community. A cutoff of 19.5 yielded the highest sensitivity (69.7%) and specificity (75.5%); the Youden index (sensitivity + specificity − 1) was 0.452. Therefore, when the COPD-SQ score was ≥19.5, it could be used as a preselection tool in primary-care settings (Figure 3).

### 4.4. Investigation of Reasons for Missed Diagnosis and Missed Treatment of COPD

**Figure 4:**
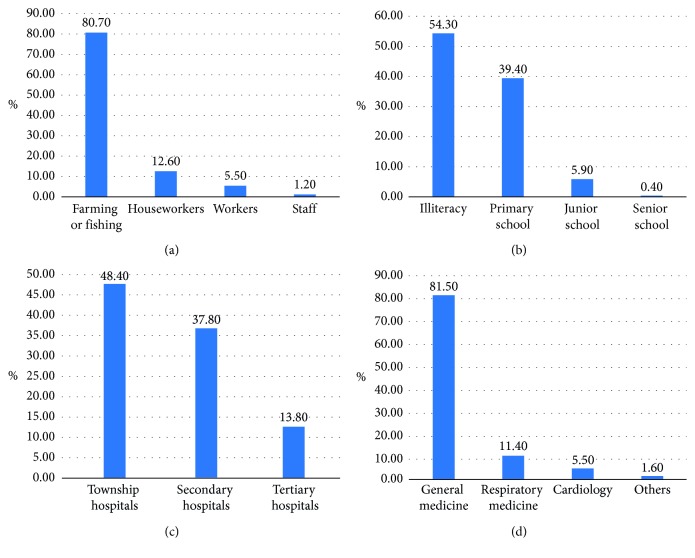
General surveys of patients with COPD. (a) COPD occupational survey. (b) COPD educational level survey. (c) COPD patients in the highest hospitals. (d) COPD patients in the highest departments.

- Among 254 listed individuals diagnosed with COPD, only 10 had a history of COPD and only 35 had a previous diagnosis of pneumonia or bronchitis. These data revealed that the rate of missed diagnosis of COPD in the Ningbo islands was extremely high (96.1%).
- Classification of pulmonary function in patients with COPD is given in Table 3.
- In total, 254 individuals had COPD (GOLD stage I: *n*=104 (40.9%); GOLD stage II: *n*=106 (41.7%); GOLD stage III: *n*=36 (14.2%); and GOLD stage IV: *n*=8 (3.2%)).
- Investigation of the general situation regarding COPD.
- Statistics regarding education level, occupation, and medical treatment are presented in Figure 4.

## 5. Discussion

### 5.1. Analysis of the Prevalence of COPD

Our survey found that the prevalence of COPD among residents ≥60 years of age in the Ningbo island area was as high as 18.5%. A previous study pointed out that the prevalence of COPD in Chinese individuals ≥40 years of age was 9.9%, and the prevalence of COPD increased with age, from 3.2% in those 40–49 years to 20.3% in those >70 years of age [11]. The CPH study reported that the prevalence of COPD increased with age in the same region and sex. In the same age group and region, the prevalence was higher in men than in women. In the same age group and sex, the prevalence of COPD in rural areas was higher than in urban areas [5]. This was consistent with our statistical data (Figure 2). In the same age group, prevalence was higher in men than in women. The prevalence of COPD increased with age in males and females.

### 5.2. Analysis of Risk Factors for COPD

#### 5.2.1. Sex

The survey results revealed that the prevalence of COPD in males (28.9%) was higher than in females (8.3%). However, according to multivariate logistic regression analysis, sex was not an independent factor for COPD. It may be related to the high smoking rate in males. Of the 682 males in the data set, 551 (80.8%) smoked; of the 689 females in the data set, only 21 (3.0%) smoked.

#### 5.2.2. Age

The survey results revealed that age was an independent risk factor for COPD. With increases in age, organ structure declines and changes in thoracic structure occur, in addition to airway remodeling under the stimulation of long-term chronic inflammatory factors, which ultimately culminate in the occurrence of COPD.

#### 5.2.3. Smoking Pack-Years and Exposure to Biomass Smoke from Cooking

The survey revealed that smoking history and exposure to biomass smoke from cooking were not independent risk factors for COPD.

Previous studies have reported that the high prevalence of smoking and biomass fuel use were the primary causes of the high incidence of COPD [12]. Our investigation found that the prevalence of COPD with a history of smoking was higher than that without smoking. Due to long-term exposure to tar and nicotine fumes in cigarette smoke, inflammatory cells, especially neutrophils and macrophages, are recruited and activated, releasing serine and matrix metalloproteinases, further activating the oxidative stress response that helps to repair and remove foreign substances. Emphysema occurs when the destruction of extracellular matrix and cell death exceed their repair capacity. These factors can accelerate the development of COPD [13].

The prevalence of COPD with exposure to biomass smoke from cooking was lower than that of no exposure to biomass smoke. This was inconsistent with previous conclusions that air pollution caused by long-term exposure to biofuels, especially solid biofuels, is the main cause of COPD [14]. Possible reasons include the following. In this survey, the sample size and the number of years of exposure to biofuels were too small to cause inflammatory damage to the airway. Second, among 997 individuals exposed to biofuels, 62.6% were females, among whom the prevalence of COPD was only 8.3%, which may be related to the high biofuel exposure rate of females. Finally, among males diagnosed with COPD, 32 had a history of dust exposure, such as burning tiles and mining, accounting for 16.2% of the males with COPD. Air and dust pollution, combined with poor ventilation and long-term deposition in the respiratory tract, could cause chronic and long-term airway remodeling and contribute to the development of COPD [15].

#### 5.2.4. Family History of Respiratory Diseases

The survey results demonstrated that there was no correlation between family history of respiratory diseases and the prevalence of COPD. This may have been related to the insufficient sample size of the survey, or with aging, which negatively affects memory recall in the elderly regarding family history of respiratory diseases and low levels of medical care in the past.

#### 5.2.5. Cough and Dyspnea

The prevalence of COPD in individuals with cough symptoms was significantly higher than in those without cough symptoms: the higher the degree of dyspnea, the higher the prevalence of COPD.

### 5.3. COPD-SQ

The COPD-SQ consisted of seven items addressing the following: age, smoking pack-years, BMI, cough, dyspnea, family history of respiratory diseases, and exposure to biomass smoke from cooking. Results of the survey revealed that a cutoff of 16 yielded the best sensitivity (72.6%) and specificity (78.0%); therefore, scores >16 could be used to diagnose COPD [16]. Our data revealed a sensitivity of 89.4% and a specificity of 43.7%. Receiver operating characteristic (ROC) curve analysis yielded a sensitivity of 69.7% and a specificity of 75.5% at a cutoff of 19.5, suggesting that the COPD-SQ could be used as a preselection screening tool in primary care settings. Of course, this was inconsistent with studies reporting a COPD-SQ score >16. This may be related to the fact that all of the individuals we surveyed were >60 years of age, and the basic age score was >8; therefore, the cutoff value for COPD-SQ score was high. However, the age of survey populations reported in the literature is >40 years, and the statistical age span is wide, so it was feasible to assign the score. Therefore, according to the results of our survey, the cutoff COPD-SQ score (>19.5) was more suitable for older individuals (≥60 years) and could be used as a community COPD screening tool in the elderly.

### 5.4. Analysis of Missed Diagnosis of COPD

COPD occurs mostly in the elderly. When patients do not exhibit the corresponding symptoms or have atypical symptoms, they can experience airflow limitation, which leads to missed diagnosis. Among the 254 individuals diagnosed with COPD in this study, only 10 had a history of COPD and only 35 had a previous diagnosis of pneumonia or bronchitis. Among the 10 patients, only one had undergone standardized treatment. These data reveal that the rate of missed diagnosis of COPD in the Ningbo island area was 96.1%. Only 13 (5.1%) underwent pulmonary function testing and 241 (94.9%) did not. This figure was significantly higher than the rate of missed diagnosis of COPD reported in another study investigating Ningbo (82.3%) [17]. We analyzed the reasons for missed diagnosis of COPD. The education level of the patients in the island area was generally low and occupations were relatively fixed. Previous studies have reported [18] that approximately 96.4% of COPD patients in rural areas have no knowledge of COPD before diagnosis and approximately 32.1% do not know that smoking is a risk factor for COPD. As shown in Figure 4, the rates of illiteracy, primary school, junior high school, and senior high school education were 54.3%, 39.4%, 5.9%, and 0.4%, respectively. The rates of farming or fishing, houseworkers, workers, and staff were 80.7%, 12.6%, 5.5%, and 1.2%, respectively. Low education would affect COPD education and COPD self-management. Farmers or fishermen worked mainly in the open air, and the work is monotonous and dull; consequently, the smoking rate would increase accordingly, which plays an important role in COPD airway inflammation [19]. The island area is remote, the traffic is inconvenient, and the economic level is relatively low. As shown in Figure 4, the proportion of COPD patients in the highest hospitals, in township hospitals, secondary hospitals, and tertiary hospitals, was 48.4%, 37.8%, and 13.8%, respectively. In general medicine, respiratory medicine, cardiology, and others were 81.5%, 11.4%, 5.5%, and 1.6%, respectively. Most of the COPD patients went to the general medicine department of township hospitals but failed to reach the respiratory specialty of tertiary hospitals, which resulted in the missed diagnosis of COPD. Primary hospitals had poor medical conditions, lack of pulmonary function equipment, and insufficient supply of drugs to treat COPD. Most drugs were expectorants and inhalants and were difficult to obtain. Community doctors were not specialists in respiratory medicine, and they did not have sufficient awareness of COPD to properly diagnose it. Often, the patients were misdiagnosed with bronchitis or pneumonia in the course of an acute attack.

### 5.5. Analysis of Missed Treatment of COPD

Due to lack of attention to the symptoms of COPD, most patients were not diagnosed with COPD. During the stable period, doctors did not prescribe drugs and did not perform the corresponding treatment. Among the 10 patients who had been diagnosed with COPD, the reasons for the lack of long-term standardized treatment included ineffectiveness; patients not able to effectively grasp the method of drug inhalation; patients stopped taking drugs when they felt better; difficulty in obtaining drugs; and patients not received treatment for economic reasons.

### 5.6. GOLD Classification of Pulmonary Function in Patients with COPD

This survey showed that patients with GOLD I and II COPD comprised up to 82.7% of the sample. A previous epidemiological survey reported that 70.7% of patients with COPD belonged to the GOLD I and II categories [20]. Individuals with low-grade COPD often lack the corresponding symptoms and are often misdiagnosed with chronic bronchitis without long-term standardized treatment. COPD progresses slowly, and its early symptoms can be atypical or neglected. Once obvious symptoms emerge, most patients are already in the middle and late stages of the disease. Studies have shown that patients with GOLD II experience the most rapid decline in pulmonary function [21]. With the increase in pulmonary function classification, the mean values of mMRC and CAT scores also increase. This indicates that the mMRC could be used to evaluate the degree of dyspnea in the daily life of COPD patients. Of course, in the absence of a spirometer, whether mMRC and CAT can be used to diagnose or grade COPD requires further verification.

### 5.7. Sample Loss in This Survey

Of course, if the survey population we choose is ≥60 years of age, it will lead to missed diagnosis of COPD in people younger than 60 years old. However, there are some reasons. According to our presurveys, the prevalence of people ≥60 years of age is much higher than that of people <60 years of age and most of the young people go out to work and study. Furthermore, the purpose of this survey is to know the prevalence and missed diagnosis of COPD in the elderly.

Then, we used a census method that we chose all the people ≥60 years of age on this island, instead of conducting a random survey. With the help of local disease control and village chiefs, through overall mobilization, we posted banners, health checkup methods, and pursued individual mobilization according to personnel lists to improve compliance. However, some residents did not participate in the survey because of physical reasons, some went fishing or went to work, and some lived outside the island with their children. All of these reasons may have contributed to errors in this survey.

Overall, the prevalence of COPD was 18.5% among residents >60 years of age in an island area of Ningbo. Age and smoking were independent risk factors for COPD. Only 10 individuals had a history of COPD, and the rate of missed diagnosis was 96.1%. The prevalence of COPD and missed diagnosis rate of COPD among the elderly in the Ningbo island area was very high. Community screening and pulmonary function testing were helpful for early diagnosis of COPD.

To conduct this survey, we hope that the government and people will be aware of the high prevalence of COPD and pay as much attention to COPD as to hypertension, diabetes, and coronary heart disease. On the one hand, we used community screening to provide early warning of COPD in the community. On the other hand, pulmonary function testing was used to confirm the diagnosis of COPD.

It was said in an article that early detection, diagnosis, and intervention should be the main focus of prevention and treatment of COPD in China [22]. Therefore, we may improve the earlier detection and treatment of COPD through the following measures.

First of all, the high prevalence of COPD should be reported on TV and newspapers, so that the public could improve their understanding of COPD. At the same time, we should promote COPD-SQ in the community so that people can warn COPD. Secondly, it is recommended to the government that the pulmonary function testing is included in the scope of medical insurance in the annual physical examination. Finally, in the earlier treatment, expectorants, inhalants, and other drugs will be included in the scope of community treatment drugs to provide patients with stable treatment. As well, community doctors should be trained to improve the diagnosis and treatment of COPD.

## Figures and Tables

**Table 1:** Comparison of prevalence among individuals with different factors.

	Number of cases	Prevalence rate (*n* (%))	*χ* ^2^ value	*P* value
Gender			96.478	<0.01
Men	682	197 (28.9)		
Women	689	57 (8.3)		
Age			55.189	<0.01
60–69 years	786	99 (12.6)		
70–79 years	475	113 (23.8)		
≥80 years	110	42 (38.2)		
Smoking			133.722	<0.01
Yes	572	188 (32.9)		
No	799	66 (8.3)		
Smoking (pack-years)			150.310	<0.01
0	799	66 (8.3)		
1–14.9	66	12 (18.2)		
15–29.9	106	31 (29.1)		
≥30	400	145 (36.1)		
BMI			2.654	>0.01
<18.5	56	14 (25.0)		
18.5–23.9	692	133 (19.2)		
24.0–27.9	480	81 (16.9)		
≥28	143	26 (18.2)		
Family history of respiratory diseases			2.659	>0.01
Yes	135	32 (23.7)		
No	1236	222 (18.0)		
Exposure to biomass smoke from cooking			Unable to judge	Unable to judge
Yes	997	155 (15.6)		
No	374	99 (26.5)		
Cough			129.282	<0.01
Yes	458	162 (35.4)		
No	913	92 (10.1)		
Dyspnea			114.045	>0.01
Level 1	1024	128 (12.5)		
Level 2	280	90 (32.1)		
Levels 3–5	67	36 (53.7)		

**Table 2:** Multivariate logistic regression analysis of residents with COPD.

Variable	B	SE	Wald	Significant	OR value	95% CI
Sex	0.459	0.264	3.023	0.082	1.582	(0.943, 2.655)
Age	0.078	0.011	48.303	<0.01	1.082	(1.058, 1.106)
Smoking	−1.359	0.250	29.441	<0.01	0.257	(0.157, 0.420)

**Table 3:** The relationship between factors in different sex GOLD classification.

	Men					Women				
	Total *n* = 682	NO COPD *n* = 485	GOLD stage I *n* = 82	GOLD stage II *n* = 83	GOLD stage III-IV *n* = 32	Total *n* = 689	No COPD *n* = 632	GOLD stage I *n* = 22	GOLD stage II *n* = 23	GOLD stage III-IV *n* = 12
Age (years)	69.7 ± 6.7	68.9 ± 6.3	70.5 ± 6.9	72.1 ± 6.8	73.5 ± 7.2	68.5 ± 6.0	68.1 ± 5.8	74.9 ± 6.7	71.0 ± 5.6	71.8 ± 6.4
60–69	366 (53.7%)	288 (59.4%)	40 (48.8%)	31 (37.3%)	8 (25.0%)	419 (60.8%)	399 (63.1%)	4 (18.2%)	12 (52.2%)	4 (33.3%)
70–79	246 (36.1%)	159 (32.8%)	30 (36.6%)	39 (47.0%)	17 (53.1%)	230 (33.4%)	203 (32.1%)	11 (50.0%)	9 (39.1%)	7 (58.3%)
≥80	70 (10.3%)	38 (7.8%)	12 (14.6%)	13 (15.7%)	7 (21.9%)	40 (5.8%)	30 (4.7%)	7 (31.8%)	2 (8.7%)	1 (8.3%)

Smoking										
Yes	551 (80.8%)	370 (76.3%)	70 (85.4%)	79 (95.2%)	32 (100.0%)	21 (3.0%)	14 (2.2%)	1 (4.5%)	4 (17.4%)	2 (16.7%)
No	131 (19.2%)	115 (23.7%)	12 (14.6%)	4 (4.8%)	0 (0.0%)	668 (97.0%)	618 (97.8%)	21 (95.5%)	19 (82.6%)	10 (83.3%)

Smoking (pack-years)										
0	131 (19.2%)	115 (23.7%)	12 (14.6%)	4 (4.8%)	0 (0.0%)	668 (97.0%)	618 (97.8%)	21 (95.4%)	19 (82.6%)	10 (83.3%)
1–14.9	59 (8.7%)	48 (9.9%)	5 (6.1%)	6 (7.2%)	0 (0.0%)	7 (1.0%)	6 (0.9%)	0 (0.0%)	0 (0.0%)	1 (8.3%)
15.0–29.9	100 (14.7%)	70 (14.4%)	7 (8.5%)	17 (20.5%)	6 (18.8%)	6 (0.8%)	5 (0.8%)	0 (0.0%)	1 (4.3%)	0 (0.0%)
≥30	392 (57.5%)	252 (52.0%)	58 (70.7%)	56 (67.5%)	26 (81.2%)	8 (1.2%)	3 (0.5%)	1 (4.5%)	3 (13.0%)	1 (8.3%)

BMI										
<18.5	28 (4.1%)	19 (3.9%)	1 (1.2%)	4 (4.8%)	4 (12.5%)	28 (4.1%)	23 (3.6%)	3 (13.6%)	2 (8.7%)	0 (0.0%)
18.5–23.9	372 (54.5%)	262 (54.0%)	49 (59.8%)	40 (48.2%)	21 (65.6%)	320 (46.4%)	297 (47.0%)	6 (27.3%)	11 (47.8%)	6 (50.0%)
24.0–27.9	231 (33.9%)	167 (34.4%)	28 (34.1%)	33 (39.8%)	3 (9.4%)	249 (36.1%)	232 (36.7%)	8 (36.4%)	7 (30.4%)	2 (16.7%)
≥28.0	51 (7.5%)	37 (7.6%)	4 (4.9%)	6 (7.2%)	4 (12.5%)	92 (13.4%)	80 (12.7%)	5 (22.7%)	3 (13.0%)	4 (33.3%)

Family history of respiratory diseases										
Yes	64 (9.4%)	38 (7.8%)	6 (7.3%)	13 (15.7%)	7 (21.9%)	71 (10.3%)	65 (10.3%)	2 (9.1%)	2 (8.7%)	2 (16.7%)
No	618 (90.6%)	447 (92.2%)	76 (92.7%)	70 (84.3%)	25 (78.1%)	618 (89.7%)	567 (89.7%)	20 (90.9%)	21 (91.3%)	10 (83.3%)

Exposure to biomass smoke from cooking										
Yes	373 (54.7%)	269 (55.5%)	43 (52.4%)	42 (50.6%)	19 (59.4%)	624 (90.6%)	573 (90.7%)	19 (86.4%)	21 (91.3%)	11 (91.7%)
No	309 (45.3%)	216 (44.5%)	39 (47.6%)	41 (49.4%)	13 (40.6%)	65 (9.4%)	59 (9.3%)	3 (13.6%)	2 (8.7%)	1 (8.3%)

Cough										
Yes	284 (41.6%)	154 (31.8%)	49 (59.8%)	54 (65.0%)	27 (84.4%)	174 (25.3%)	142 (22.5%)	9 (40.9%)	15 (65.2%)	8 (66.7%)
No	398 (58.4%)	331 (68.2%)	33 (40.2%)	29 (35.0%)	5 (15.6%)	515 (74.7%)	490 (77.5%)	13 (59.1%)	8 (34.8%)	4 (33.3%)

Dyspnea										
Level 1	523 (76.7%)	423 (87.2%)	59 (72.0%)	34 (41.0%)	7 (21.9%)	501 (72.7%)	473 (74.8%)	17 (77.3%)	9 (39.1%)	2 (16.7%)
Level 2	126 (18.5%)	52 (10.7%)	18 (22.0%)	40 (48.2%)	16 (50.0%)	154 (22.4%)	138 (21.8%)	2 (9.1%)	9 (39.1%)	5 (41.7%)
Level 3–5	33 (4.8%)	10 (2.1%)	5 (6.1%)	9 (10.8%)	9 (28.1%)	34 (4.9%)	21 (3.3%)	3 (13.6%)	5 (21.8%)	5 (41.7%)

mMRC	—	—	1.3 ± 0.9	1.8 ± 1.0	2.4 ± 1.3	—	—	1.3 ± 1.1	1.4 ± 1.1	2.5 ± 1.6
CAT	—	—	6.1 ± 5.9	7.5 ± 6.1	13.4 ± 8.2	—	—	6.2 ± 5.5	6.8 ± 5.3	10.7 ± 8.5
FEV_1_ (L)	2.3 ± 0.6	2.5 ± 0.5	2.2 ± 0.4	1.6 ± 0.3	0.9 ± 0.3	1.8 ± 0.4	1.9 ± 0.4	1.5 ± 0.4	1.1 ± 0.2	0.7 ± 0.1
FVC (L)	3.1 ± 0.7	3.1 ± 0.6	3.4 ± 0.6	2.6 ± 0.5	2.0 ± 0.6	2.2 ± 0.5	2.3 ± 0.5	2.3 ± 0.5	1.8 ± 0.3	1.2 ± 0.2

## Data Availability

The data used to support the findings of this study are available from the corresponding author upon request.
